# Patient perceived barriers to surgical follow-up: Study of 6-month post-operative trichiasis surgery follow-up in Tanzania

**DOI:** 10.1371/journal.pone.0247994

**Published:** 2021-03-19

**Authors:** Michael Saheb Kashaf, Meraf A. Wolle, Beatriz E. Muñoz, Harran Mkocha, Nicodemus Funga, Catherine Gracewello, Sheila K. West

**Affiliations:** 1 Dana Center for Preventive Ophthalmology, Wilmer Eye Institute, Johns Hopkins University, Baltimore, MD, United States of America; 2 Kongwa Trachoma Project, Kongwa, United Republic of Tanzania; Helen Keller International, UNITED KINGDOM

## Abstract

**Background:**

Post-surgical follow-up is a challenge in low- and middle-income countries. Understanding barriers to trachomatous trichiasis (TT) surgical follow-up can inform program improvements. In this study, patient perceived barriers and enabling factors to follow-up after TT surgery are identified.

**Methods:**

A longitudinal study was carried out in a community-based cohort of persons who received TT surgery in Bahi district, Tanzania. Questionnaires were administered before TT surgery and again after the scheduled 6-month follow-up. Those who did not return were examined at their homes.

**Results:**

At baseline, 852 participants were enrolled. Of these, 633 (74%) returned at 6 months and 128 (15%) did not and were interviewed at home. Prior to surgery, attenders were more likely to report familiarity with a community health worker (CHW) (22% vs. 14%; p = 0.01) and less likely to state that time constraints are a potential reason for failure to follow-up (66% vs. 74%; p = .04). At follow-up, non-attenders were more likely to endorse barriers pertaining to knowledge about the need for follow-up, lack of transportation, and satisfaction with surgery. There was no difference in post-operative TT between attenders and non-attenders (23% vs. 18% respectively; p = 0.25).

**Conclusions:**

The outcome of surgery was not a barrier to follow-up. However, better integration of CHWs into their communities and work at coordinating post-surgical care may improve follow-up rates. Moreover, provision of transportation and implementation of effective reminder systems may address patient-perceived barriers to improve follow-up.

## Introduction

Post-operative follow-up addresses surgical complications and allows for monitoring of effectiveness. The Global Burden of Surgical Disease Working Group has recommended that global surgery initiatives establish a clear mechanism for post-surgical follow-up [1]. In the context of global health programs, post-surgical follow-up is also an ethical obligation [2]. Gaps in follow-up have come under increasing scrutiny as an important shortcoming in the delivery of global health surgery programs [3, 4]. This has necessitated research to identify barriers and enabling factors influencing post-operative follow-up.

Trachomatous trichiasis (TT) is a late complication of trachoma, a chronic conjunctivitis caused by repeated ocular infections with *Chlamydia trachomatis*. Surgery can correct TT and prevent blinding complications. The World Health Organization (WHO) has identified trichiasis surgery as a core element of its trachoma elimination strategy [5]. Randomized controlled trials have demonstrated the safety and efficacy of trichiasis surgery [6, 7].

Post-operative trichiasis can be as low as 6% at 1 year, in the setting of proficient provider training and certification [8]. However, most programs report higher rates, which have been attributed to surgical skill and severity of pre-operative TT [8–10]. A WHO technical working group recommended 6-month follow-up of post-surgical cases to monitor outcomes [11].

Despite significant risk of post-operative TT, rates of post-surgical follow-up remain low. In a large post-surgical cohort in Ethiopia, only 21.8% of patients were seen at 6 months [12]. Most patients present at 1 day, and some at 1–2 weeks, but there is a steep drop off for follow-up at 3–6 months [12]. The reasons for poor post-surgical follow-up are not known, though studies of patient perceived barriers to initial receipt of TT surgery suggest that logistical factors, patient education, patient perceptions about TT management, and competing familial and work responsibilities may be important [13]. Moreover, there is concern that high rates of adverse outcomes may be a factor.

The aim of this study is to investigate patient perceived barriers and enabling factors associated with 6-month follow-up after TT surgery in the Bahi district of Tanzania. There are several specific objectives: First, to explore participant characteristics associated with post-surgical follow-up; Second, to investigate patient perceived barriers and enabling factors related to follow-up; and third, to assess whether post-operative TT rates differ between those who did and those who did not return for their 6-month follow-up visit.

## Materials and methods

### Study design and participants

A prospective study of trichiasis patients who underwent surgery from 1/29/2018 to 01/05/2019 was conducted in Bahi district, Tanzania. Bahi is one of the districts targeted by the Tanzania National Tropical Disease Control Program for special community-based TT identification and surgery initiatives. In each village, 3–4 community health workers (CHWs) were assigned to go house-to-house to screen for TT cases, list them as surgical candidates, and ensure they presented at a surgical camp. At the camp, cases were examined by a surgeon for operable TT and offered surgery.

A trained grader performed an ocular examination using a 2.5x loupe and flashlight to assess the presence and severity of TT prior to surgery. Graders evaluated TT as mild, moderate, or severe (Table 1).

**Table 1:** Grading criteria defining baseline TT severity.

Grade	Criteria
Mild TT	1. 1–5 eyelashes touching the globe without any history of epilation (removal of lashes with forceps), or
	2. <1/3 of the eyelid epilated, with no lashes touching the globe
Moderate TT	1. 6–9 eyelashes touching the globe without any history of epilation, or
	2. 1–5 eyelashes touching globe and <1/3 of eyelid epilated
Severe TT	1. 6–9 eyelashes touching globe and any epilation, or
	2. ≥10 eyelashes touching globe, regardless of epilation status, or
	3. ≥1/3 of eyelid epilated

A trained interviewer collected demographic information and administered the pre-surgery questionnaire in a local language. Following surgery, participants were reminded by CHWs to present for 6-month surgical follow-up at a central location in each participating village, and reminded again immediately prior to the visit. Participants attending the 6-month follow-up visit were administered a post-surgery questionnaire designed for those presenting. For participants failing to attend, contact was attempted at home, and a questionnaire designed for non-attenders was administered. Another visit by the study team trichiasis grader was undertaken 4 to 6 months after surgery to assess the presence of post-operative trichiasis, defined as at least one eyelash touching the globe and/or evidence of epilation.

This study was approved by the Johns Hopkins Institutional Review Board and by the Ethics Committee of the Tanzania National Institute for Medical Research. It adhered to the declaration of Helsinki. All participants provided written informed consent in Swahili or the local languages.

### Survey instruments

We used three questionnaires, one for pre-surgery participants and two for use after surgery, based on a previously developed instrument designed to assess barriers and enabling factors for acceptance of TT surgery [13]. All three questionnaires used pre-coded and open-ended questions to elicit potential reasons as to why “persons in this village who had surgery did not come back for follow-up visits”. There were two post-surgery questionnaires, one for those patients who self-presented for follow-up and the other for patients not presenting (non-attenders). The post-surgery questionnaire used among non-attenders elicited personal barriers to follow-up and surveyed participants regarding their suggestions for improving follow-up.

### Sample size and data analyses

We assumed that 50% of the sample would be attenders, with an overall post-operative trichiasis rate of 20%. With alpha = 0.05, and 80% power to detect an absolute difference of 10% in post-operative TT rates, we projected a required sample of 500 participants. With loss to follow-up of 15%, we planned to enroll 575 participants. Given the number of patients seen per day in the camps, we planned enrollment to occur over a 12-month period.

All data were entered into a Microsoft Access database. Freeform answers were translated into English by study personnel masked to whether the answers were from attenders or non-attenders. Responses were grouped into categories according to key words. All statistical analyses were conducted on SAS 9.4 software (SAS, Cary, NC).

Contingency tables were used to assess differences between attenders and non-attenders. Chi-square and Fischer’s exact testing, when indicated, were used to test the significance of differences.

## Results

**Fig 1:**
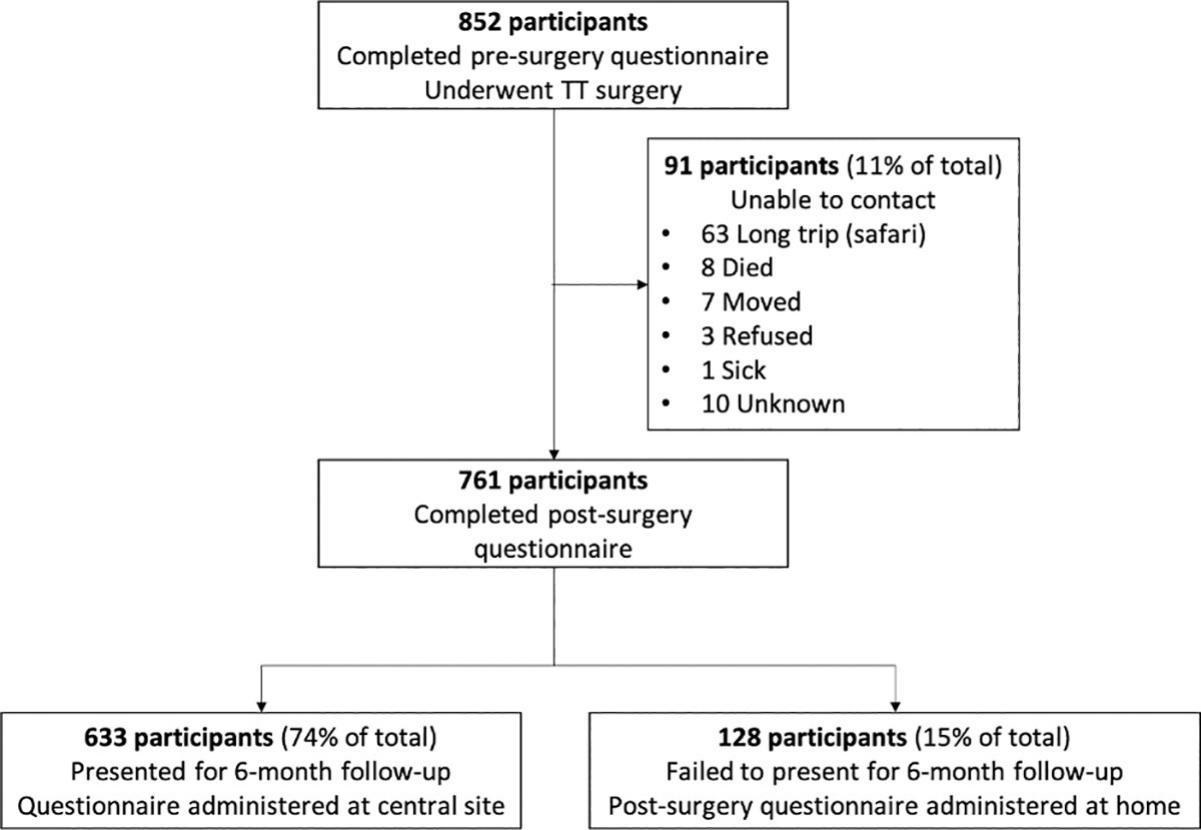
Flow of participants.

At baseline, 852 participants with TT, referred by the district network of CHWs, were consented and enrolled. These participants all completed the pre-surgery questionnaire at designated village coordination centers and underwent TT surgery. There were 761 (89%) participants who either returned for follow-up or were contacted at home at 6 months; 91 (11%) could not be contacted. Of the 761 participants, 633 (83%) presented for 6-month follow-up and completed a post-surgery questionnaire. The remaining 128 (17%) were contacted at home by study personnel and administered the post-surgery questionnaire (Fig 1). The most common reasons patients could not be contacted were extended travel (69%), death (9%), and relocation (8%). These reasons were determined by querying community members or other household members. Participants who could not be contacted were more likely to be male (31% vs. 20%, p = 0.001) and had been residents of the community for a shorter time (46 years vs. 51 years, p = 0.03). All other known characteristics were similar.

Patients who presented for follow-up were more likely to report strong familiarity with a CHW at baseline (22% vs 14.2%, p = 0.013). Attenders were also more likely to endorse satisfaction with surgery at follow-up (97.9% vs. 92.2%, p = 0.0005), although overall satisfaction was high. There were no other differences elicited at baseline between attenders and non-attenders (Table 2).

**Table 2:** Participant characteristics at baseline and follow-up.

Characteristic	Came to the central site at 6 months N = 633	Did not come to the central site N = 219	p-value
Age in years (Mean (SD))	66.5 (15.3)	67.6 (18.9)	0.435
Female	500/633 (79.0)	173/219 (79.0)	0.998
Duration of residency in community (Years) (Mean (SD))	51.1 (19.7)	48.3 (24.6)	0.119
TT duration (Years)	7.1 (6.8)	8.0 (7.2)	0.115
Reported previous TT surgery	88/632 (13.9)	33/219 (15.1)	0.676
Severe TT prior to surgery	292/633 (46.1)	102/219 (46.6)	0.909
Bilateral TT surgery	439/633 (69.1)	139/218 (63.8)	0.127
**Report knowing one of the CHWs at baseline survey**	**139/633 (22.0)**	**31/219 (14.2)**	**0.013**
**Report satisfaction with surgery at follow-up survey**	**617/630 (97.9)**	**118/128 (92.2)**	**0.0005**
Any post-operative TT at follow-up#	94/414 (22.7)	19/108 (17.6)	0.251

#Available for 522 participants

Prior to surgery, most answers elicited from participants on barriers to follow-up did not differentiate those who subsequently returned for follow-up from those who did not. Non-attenders were more likely to endorse lack of time as a potential reason (73.7% vs. 66.2%, p = 0.04) (Table 3). Prior to surgery, fear was the most commonly cited reason for non-attendance in both groups. (Table 4).

**Table 3:** Pre-surgery participant responses to pre-coded potential reasons for failure to attend the 6-month post-surgical follow-up.

Potential reasons given for not attending the 6-month follow-up exam.	Came to the central site at 6 months N = 633	Did not come to the central site N = 219	p-value
Satisfied with surgery, no need to attend follow-up	525/628 (83.6)	178/218 (81.6)	0.509
**May not have time**	**416/628 (66.2)**	**160/217 (73.7)**	**0.041**
May be unhappy with results and not want to attend follow-up	386/628 (61.4)	137/218 (62.8)	0.718
May Forget	316/628 (50.3)	107/218 (49.1)	0.753
May not have received a reminder	168/628 (26.8)	60/218 (27.5)	0.825
May not have had transportation	66/628 (10.5)	22/218 (10.1)	0.862
May feel the follow-up site is too far	59/628 (9.4)	26/218 (11.9)	0.284

**Table 4:** Pre-surgery participant answers to an open-ended question about potential reasons for failure to attend the 6-month post-surgical follow-up.

Potential reasons given for not attending the 6-month follow-up exam.	Came to the central site at 6 months N = 633	Did not come to the central site N = 219	p-value
Fear	300/630 (47.6)	100/218 (45.9)	0.656
No longer a problem	105/630 (16.7)	30/218 (13.8)	0.312
Too busy	42/630 (6.7)	18/218 (8.3)	0.430
Doesn’t care	27/630 (4.9)	8/218 (3.7)	0.693
Ungrateful	26/630 (4.1)	6/218 (2.8)	0.359
No pain	22/630 (3.5)	14/218 (6.4)	0.064
No reminder	22/630 (3.5)	9/218 (4.1)	0.666
Forgets	18/630 (2.9)	5/218 (2.3)	0.659
Did not understand	11/630 (1.8)	4/218 (1.8)	0.932

At 6 months post-surgery, participants were again queried about barriers to follow-up. Non-attenders were more likely to endorse forgetting about the follow-up, not knowing where to present, distance, lack of reminders and lack of transportation as potential reasons (Table 5). Non-attenders were less likely to endorse surgical outcomes like “satisfied with surgery” and “unhappy with results” as potential reasons for failure to follow-up. (Table 5). Non-attenders were asked to provide freeform personal reasons. The three most common first reasons were: “No reminder/late reminder” (24.2%), “Forgot” (15.6%) and “Sick/in pain” (14.8%) (Table 6).

**Table 5:** Post-surgery participant responses to pre-coded potential reasons for failure to attend the 6-month post-surgical follow-up.

Potential reasons given for not attending the 6-month follow-up exam.	Came to the central site at 6 months N = 629&	Did not come to the central site N = 128*	p-value
**Satisfied with surgery**	**533/629 (84.7)**	**75/127 (59.1)**	**<0.0001**
Doesn’t have time	298/628 (47.5)	53/127 (41.7)	0.238
**Unhappy with results**	**218/629 (34.7)**	**30/127 (23.6)**	**0.016**
**No reminder**	**200/628 (31.9)**	**88/127 (69.3)**	**<0.0001**
**Forgets**	**180/629 (28.6)**	**59/127 (46.5)**	**<0.0001**
**Place is too far**	**110/624 (17.6)**	**39/125 (31.2)**	**0.0005**
**No transportation**	**43/627 (6.9)**	**35/127 (27.6)**	**<0.0001**
**Doesn’t know where to go**	**38/629 (6.0)**	**43/127 (33.9)**	**<0.0001**

& 4 participants came to the central site but did not answer the post-surgery questionnaire because of hearing problems

*Unable to contact 91 participants

Note: denominators change due to incomplete questionnaires

**Table 6:** Reasons for not coming to the central site.

Reason given	Frequency (%)
No reminder/ late reminder	31/128 (24.2)
Forgot	20/128 (15.6)
Sick / in pain	19/128 (14.8)
Away from home	13/128 (10.2)
Too far	6/128 (4.7)
Did not understand instructions	6/128 (4.7)
No longer a problem/satisfied	6/128 (4.7)
No one to bring them	6/128 (4.7)
Got there too late/too early	6/128 (4.7)
Too busy	4/128 (3.1)

First answer to open-ended question on post-surgery questionnaire for those who did not attend follow-up.

Gender differences in responses were explored. There were no differences in responses to either pre-coded or volunteered reasons for non-attendance at either time point. The only difference was that females were less likely to report knowing their CHW (OR = 0.67 (reference male), 95% CI = 0.45, 0.99, p = 0.042).

At follow-up, non-attenders were surveyed regarding their suggestions for improving attendance at the 6-month examination. The three most common suggestions were: “Provide advance notice for services, include clear instructions” (38.7%), “More health education on need for follow-up…” (14.5%) and concerns related to getting to the central follow-up site (12.1%). The most common freeform suggestions have been listed in a supplemental table (S1 Table).

Rates of post-operative TT did not differ across participants who presented for follow-up and those who failed to present (23% vs. 18% respectively; p = 0.25) (Table 2). TT severity in surgical eyes at baseline predicted risk of recurrent TT at follow-up (p < 0.0001) (Table 7). Severe TT was associated with a post-operative TT odds ratio of 2.35 (95% CI: 1.26–3.53) compared to a reference group formed by combining participants with mild and moderate severity TT.

**Table 7:** TT severity in surgical eyes at baseline and the risk of post-operative TT at follow-up.

Baseline TT severity	Number of eyes*	% post-operative TT	Test for trend
Mild	289	10.0	0.0001
Moderate	232	8.2	
Severe	355	20.0	
Total	876	13.4	

*522 participants were examined for post-operative TT

## Discussion

The preferred practice pattern for TT surgery specifies follow-up at 3–6 months to determine adverse outcomes [14]. In this cohort study, we examined patient perceived barriers and enabling factors to their attending 6-month follow-up after TT surgery in a district of Tanzania where surgical cases were reminded by CHWs to present for surgical follow-up at a central location in each village.

The strongest prospective predictor of attendance at follow-up was personal familiarity with a CHW. This was likely due to the role health workers played in delivering reminders for follow-up. Participants less familiar with their local CHWs may either have not received the notice or been less likely to heed the reminder. Indeed, at follow-up, nearly a quarter of non-attenders identified “no reminder/late reminder” as a reason for their failure to attend follow-up, the single most common first reason. The program was organized so that 3–4 CHWs in each village were assigned to identify TT cases for surgery and help with follow-up reminders. As villages can be quite de-centralized (average density for the district is 39 persons/km^2^ [15]), each CHW may have only a few cases spread over some distance. CHWs may be more likely to ensure follow-up for those they know under such circumstances. In studies of mass drug administration (MDA) of antibiotics for trachoma elimination, familiarity with CHWs was a predictor for adherence to MDA while the presence of fewer CHWs per community was a risk factor for non-participation [16]. These findings highlight the important role that CHWs play in facilitating health programs and initiatives in these communities.

It is also possible that attendance was modulated by unmeasured participant characteristics–such as native language or ethnic background, although in this district, most of the population are Wagogo and speak Kigogo, as do their CHWs. Nevertheless, unmeasured characteristics may be associated with familiarity with CHWs.

Fear was the most common potential reason for non-attendance mentioned by both attenders and non-attenders on the pre-surgery questionnaire. Fear has been consistently identified as a barrier to receiving trichiasis surgery [17, 18]. There is evidence that undertaking surgery in the village setting can help assuage patient anxiety [19]. Conducting follow-up in the same localized manner may be beneficial. Fear about the follow-up visit may reflect patient uncertainty. It is reasonable to propose that providing more information about the follow-up visit might help mitigate fear. Indeed, at the 6-month follow-up, only 2 persons cited fear as a reason to not attend, which suggests that experience with the visit helped address concerns.

At follow-up, non-attenders were more likely to endorse a number of potential reasons for failure to present for follow-up. These included, “no reminder”, “forgets”, “place is too far”, “no transportation” and “doesn’t know where to go”. These potential reasons may be viewed as indirect expressions of the participant’s personal reasons for failing to follow-up [20, 21]. The reasons cited by participants suggest a number of remedies. The provision of transportation could address the three reasons pertaining to logistics (“place is too far”, “no transportation” and “doesn’t know where to go”). Transportation for those outside a distance radius, or those who are disabled, may allow targeted assistance. In addition, broader use of patient reminders, perhaps using the increasing availability of cell phones, could address the cited failures of recollection (“no reminder” and “forgets”).

While time limitations were a prospective predictor of non-attendance, there was no difference at follow-up across attenders and non-attenders in citing “doesn’t have time”. Time limitations may have been a proxy for competing life responsibilities or of logistical challenges such as distance and disability. The specific external reasons identified at follow-up, such as transportation, may have been proxies for time. Alternatively, this may reflect social desirability biases [22]. Non-attenders at follow-up may have been unwilling to endorse time as a factor, because doing so would imply that program needs are not prioritized. Instead, these participants were more likely to endorse external factors, such as logistics and lack of a reminder.

Non-attenders responding to the post-surgery questionnaire were less likely to endorse “satisfied with surgery” and “unhappy with results” as potential reasons for failure to follow-up. There was considerable overlap between those who chose these answers, so we do not believe the dichotomy reflects different sub-groups among non-attenders. Instead, the pattern suggests that the decision to attend follow-up was more independent of perceptions regarding the outcomes of surgery. This finding argues against the hypothesis that non-attendance is driven by adverse surgical outcomes.

We did not find a difference in the rate of post-operative TT between attenders and non-attenders in our cohort. Instead, the rate of post-operative TT depended on the severity of trichiasis pre-surgery. The post-operative TT rate, around 9% in those with mild to moderate severity, was quite good and in line with the low rates reported in research trials [23]. Among those with severe pre-operative trichiasis, constituting 30% of operated eyes in this study, there was a higher rate of post-operative TT, at 20%. These findings argue for ongoing programs to ensure screening to detect TT at earlier stages and for surgery at earlier stages when post-operative TT is less likely.

There is limited evidence examining patient-reported barriers to post-surgical follow-up in low and middle-income countries [24]. In a study investigating causes of post-surgical dropout among glaucoma patients in India, loss to follow-up was associated with lower income and with misconceptions regarding surgery as a cure [25]. A study examining correlates of post-cataract surgery follow-up in rural China found that patients returning at ≥ 3 months were more likely to report higher income and recall of doctor instructions to return. The study also found that monetary compensation, advertisements and telephone reminders led to a 3-fold increase in follow-up rates [26]. Similar to our study, these reports identified reminders and patient disease knowledge as modulators of post-surgical follow-up.

Participant suggestions on improving follow-up dovetailed with reasons provided for non-attendance. The most frequent suggestion was to ensure that notifications, including clear instructions of where to go, are provided in advance. Other suggestions included assistance in travel, more health communication, and educational information from the program transmitted through CHWs. Given that we don’t really know why certain people know the CHWs better than others, it is important to standardize access to CHWs to the extent possible. This may be accomplished by ensuring that CHWs are evenly distributed in the population, understand and can speak the local languages and receive uniform training and instruction regarding facilitation of post-surgical follow-up.

A number of limitations merit consideration. First, we were unable to contact 11% (91/853) of participants recruited at baseline. The primary reason for failure to contact was participant extended travel (69% (63/91)). These participants may have differed systematically from those followed-up. However, with the low overall percentage of loss to follow-up, it is unlikely a large bias was introduced. Second, the overall rate of participant attendance at 6-month follow-up was high, at 74%. This rate was higher than that observed in several community TT surgery programs [12]. This was likely due to the influence of CHWs, who worked to encourage participant post-surgical follow-up. Indeed, CHWs received financial incentives from the Tanzania National Tropical Disease Control program to encourage greater follow-up. Thus, our findings must be interpreted in the context of a community program structured to involve local health workers. Third, we can’t ensure that participants did receive a 6-month follow-up reminder from a CHW, or that non-attenders in fact did receive notification and just reported otherwise.

This study constitutes, to our knowledge, the first empiric assessment of patient-perceived barriers and enabling factors to post-operative follow-up after trichiasis surgery. TT surgical programs may be able to expand post-surgical follow-up by better integrating CHWs in providing post-operative care, instituting timely reminder systems that include relevant information about the visit, and facilitating transportation to follow-up sites. Barriers and enabling factors identified in this African setting may be more broadly relevant to post-surgical follow-up and suggest mechanisms for improving follow-up in resource-limited settings.

## Supporting information

S1 Table Participant feedback on improving TT post-surgical follow-up. Most common freeform suggestions provided by participants who did not attend the 6-month follow-up.
(DOCX)
