# Supplementary material for: Patient perceived barriers to surgical follow-up: Study of 6-month post-operative trichiasis surgery follow-up in Tanzania
Source: PLoS One. 2021 Mar 19;16(3):e0247994. doi: 10.1371/journal.pone.0247994 (PMC7978239; doi:10.1371/journal.pone.0247994)
Supplement: S1 Table — Most common freeform suggestions provided by participants who did not attend the 6-month follow-up. (DOCX) [file pone.0247994.s001.docx]

**S1 Table. Participant feedback on improving TT post-surgical follow-up. Freeform suggestions provided by participants who did not attend the 6-month follow-up**

| **Suggestions** | **Frequency (%)** |
| --- | --- |
| Advance notice for services, include clear instructions | 48/124 (38.7) |
| More health education to families and community health workers | 18/124 (14.5) |
| No one to bring them to the central site, old people need help` | 15/124 (12.1) |
| Reminders because they forget | 12/124 (9.7) |
| Leave them alone if they don’t come | 11/124 (8.9) |
| Involve the Balozi leaders in the process | 4/124 (3.2) |
| Try to follow them at home | 4/124 (3.2) |
| No shows should receive sanctions from the government | 4/124 (3.2) |
| Difficulties getting to the site | 3/124 (2.4) |
| No one thinks about us old people | 2/124 (1.6) |
| Don’t know | 2/124 (1.6) |
| Notification have the wrong information | 1/124 (0.8) |
